# Supplementary material for: Conserving a threatened North American walnut: a chromosome-scale reference genome for butternut (Juglans cinerea)
Source: G3 (Bethesda). 2023 Sep 13;14(2):jkad189. doi: 10.1093/g3journal/jkad189 (PMC10849370; doi:10.1093/g3journal/jkad189)

A. Purged canu assembly

Bases	Bases Count	% Total	% Aligned
Basecalled	6.589e+10	100%	100%
Unmapped reads	7.918e+9	12.0%	100%
Mapped reads	5.797e+10	88.0%	100%
Softclip	2.361e+9	3.58%	100%
Aligned	5.561e+10	84.4%	100%
Matching	5.421e+10	82.3%	97.5%
Non-matching	1.404e+9	2.13%	2.52%
Insertions	4.184e+8	0.635%	0.752%
Deletions	4.940e+8	0.750%	0.888%
Mismatches	4.912e+8	0.746%	0.883%

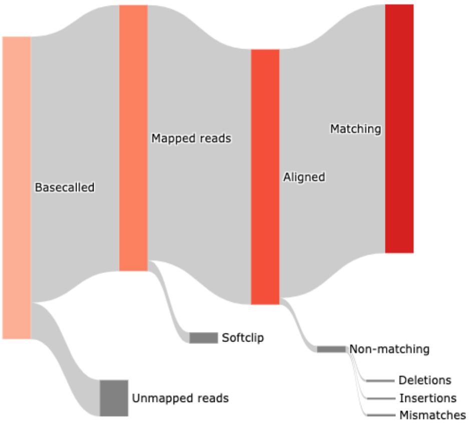

B. Scaffolded assembly after purge

Bases	Bases Count	% Total	% Aligned
Basecalled	6.589e+10	100%	100%
Unmapped reads	7.929e+9	12.0%	100%
Mapped reads	5.796e+10	88.0%	100%
Softclip	3.801e+9	5.77%	100%
Aligned	5.416e+10	82.2%	100%
Matching	5.250e+10	79.7%	96.9%
Non-matching	1.663e+9	2.52%	3.07%
Insertions	5.136e+8	0.780%	0.948%
Deletions	6.385e+8	0.969%	1.18%
Mismatches	5.111e+8	0.776%	0.944%

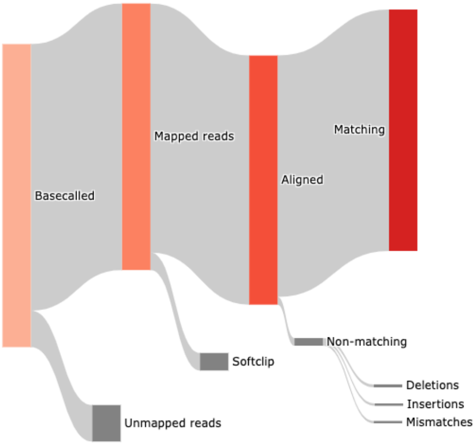

C. Pseudo chromosome assembly

Bases	Bases Count	% Total	% Aligned
Basecalled	6.589e+10	100%	100%
Unmapped reads	7.962e+9	12.1%	100%
Mapped reads	5.793e+10	87.9%	100%
Softclip	4.865e+9	7.38%	100%
Aligned	5.306e+10	80.5%	100%
Matching	5.137e+10	78.0%	96.8%
Non-matching	1.693e+9	2.57%	3.19%
Insertions	5.150e+8	0.782%	0.970%
Deletions	6.629e+8	1.01%	1.25%
Mismatches	5.150e+8	0.782%	0.971%

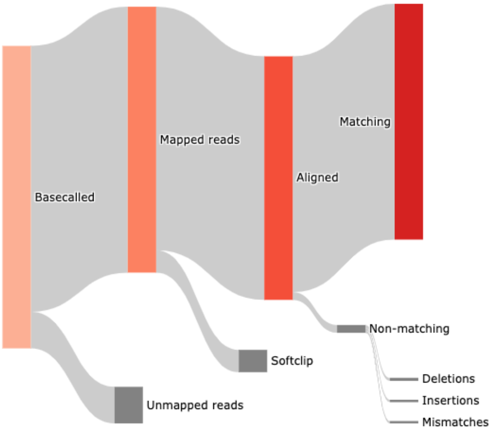

Supplement: jkad189_Supplementary_Data [file jkad189_supplementary_data.zip › Figure_S3_G3-2023-404431.pdf]
