# Supplementary figures and images for: Conserving a threatened North American walnut: a chromosome-scale reference genome for butternut (Juglans cinerea)
Source: G3 (Bethesda). 2023 Sep 13;14(2):jkad189. doi: 10.1093/g3journal/jkad189 (PMC10849370; doi:10.1093/g3journal/jkad189)

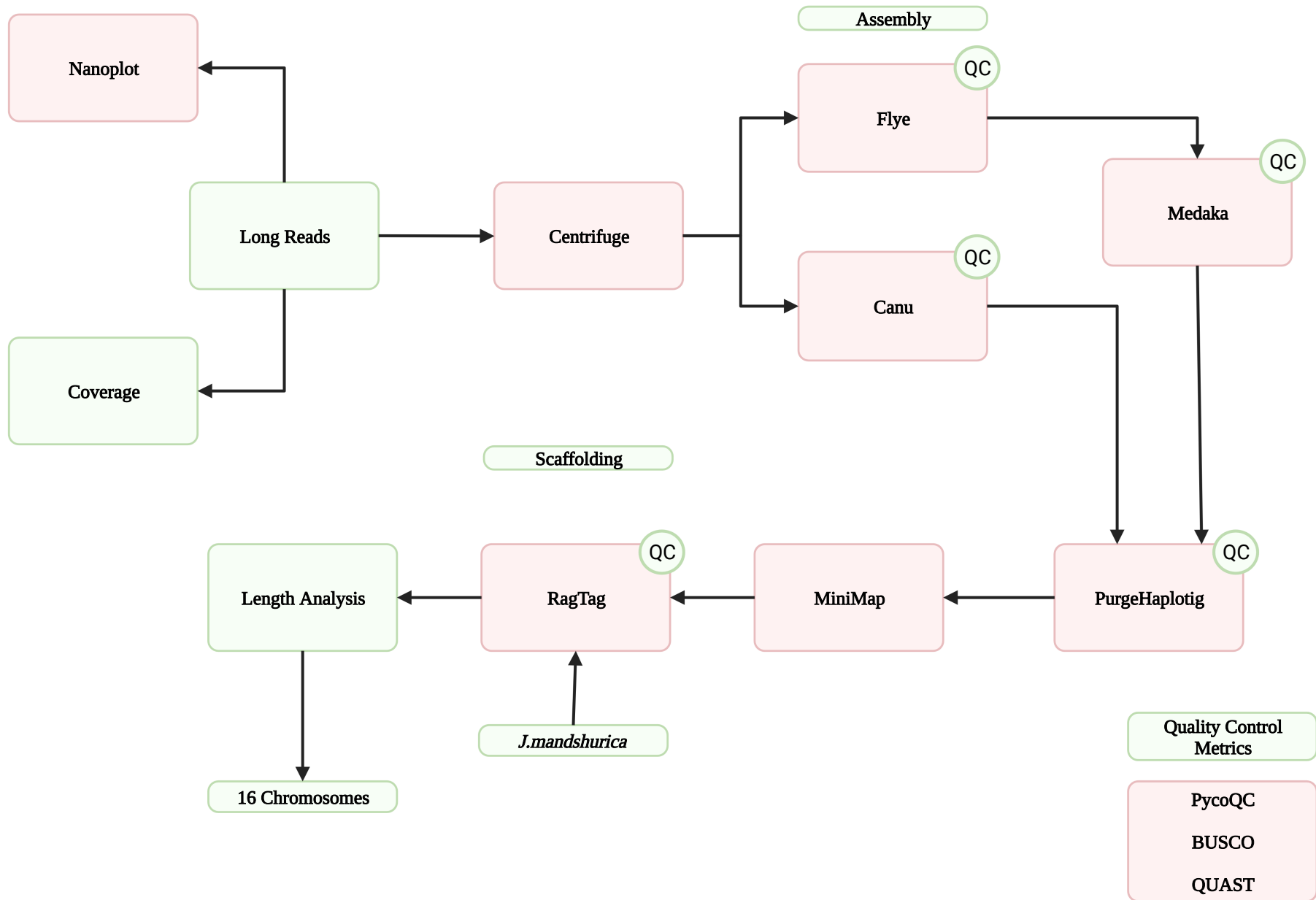

Supplement: jkad189_Supplementary_Data [file jkad189_supplementary_data.zip › Figure_S1_G3-2023-404431.pdf]

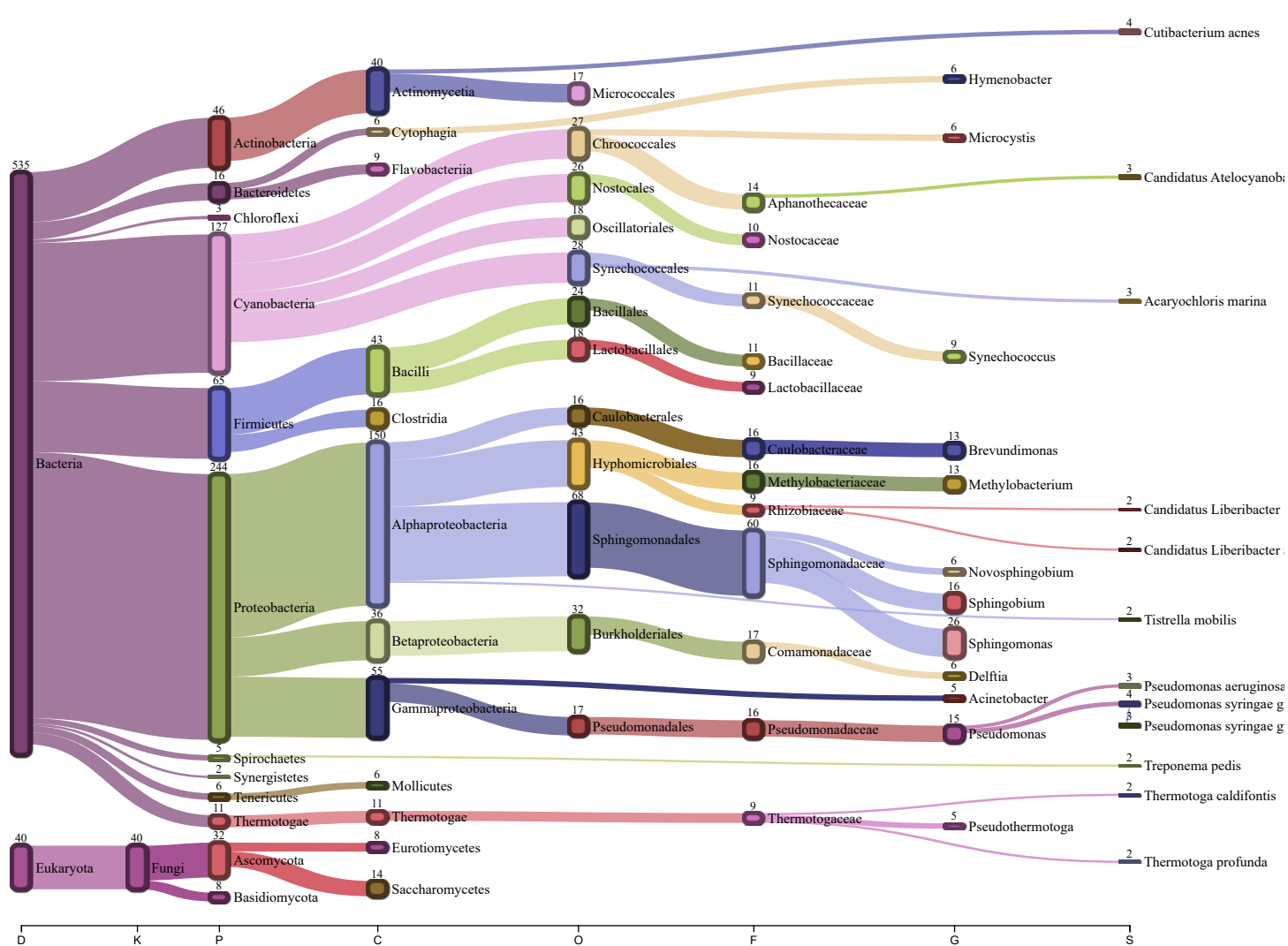

Supplement: jkad189_Supplementary_Data [file jkad189_supplementary_data.zip › Figure_S2_G3-2023-404431.pdf]

Probability of gene family expansion/contraction.

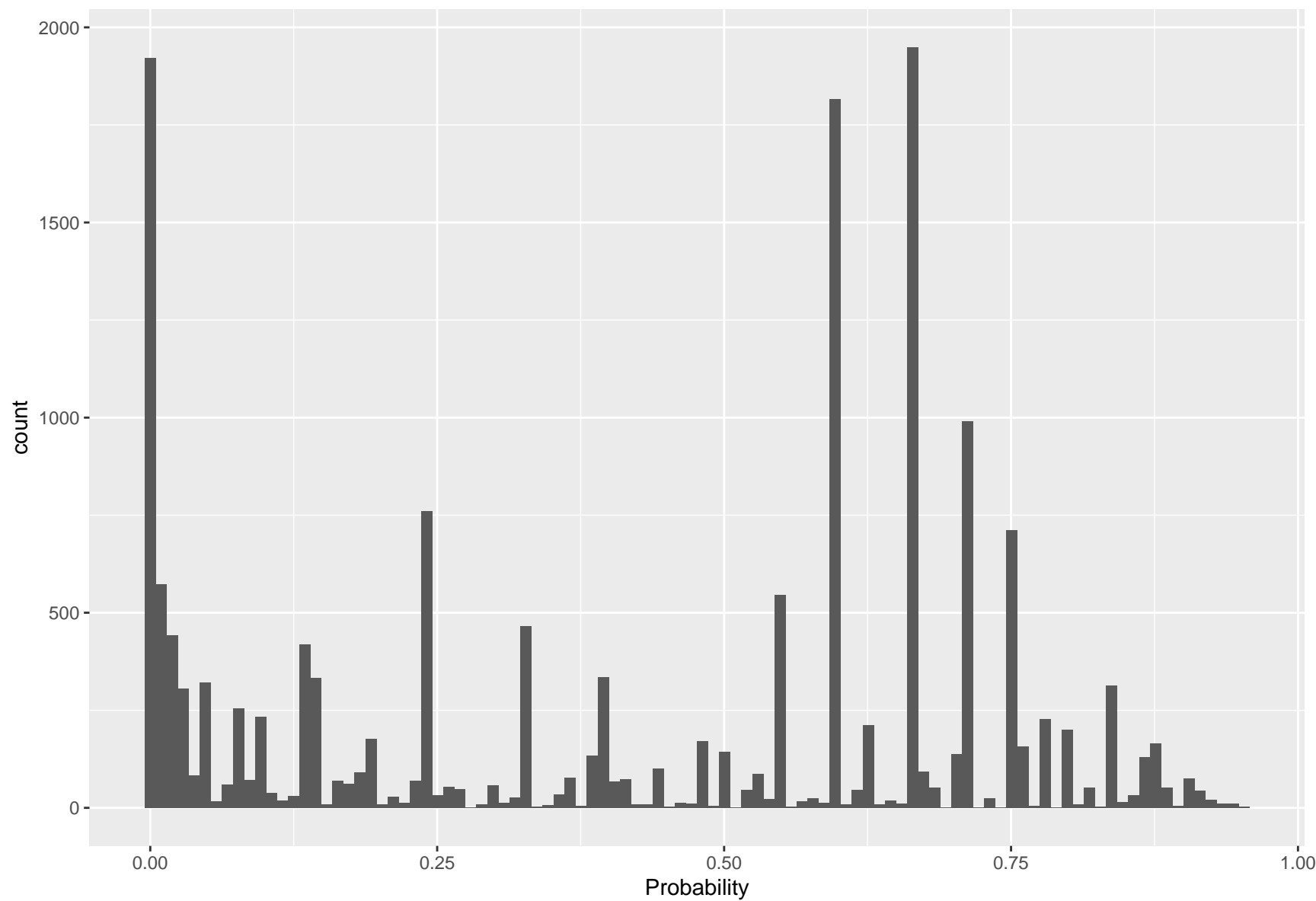

Supplement: jkad189_Supplementary_Data [file jkad189_supplementary_data.zip › Figure_S4_G3-2023-404431.pdf]
